# Adhesive threads of extraintestinal pathogenic *Escherichia coli*

**DOI:** 10.1186/1757-4749-1-22

**Published:** 2009-12-10

**Authors:** Esther-Maria Antão, Lothar H Wieler, Christa Ewers

**Affiliations:** 1 Institut für Mikrobiologie und Tierseuchen, Freie Universität Berlin, Philippstr. 13, 10115 Berlin, Germany

## Abstract

The ability to adhere to host surfaces is by far the most vital step in the successful colonization by microbial pathogens. Colonization begins with the attachment of the bacterium to receptors expressed by cells forming the lining of the mucosa. Long hair like extracellular appendages called fimbriae, produced by most Gram-negative pathogens, mediate specific attachment to the epithelial cell surface. Associated with the fimbriae is a protein called an adhesin, which directs high-affinity binding to specific cell surface components. In the last couple of years, an enormous amount of research has been undertaken that deals with understanding how bacterial pathogens adhere to host cells. *E. coli *in all probability is one of the best studied free-living organisms. A group of *E. coli *called Extraintestinal pathogenic *E. coli *(ExPEC) including both human and animal pathogens like Uropathogenic *E. coli *(UPEC), Newborn meningitic *E. coli *(NMEC) and Avian pathogenic *E. coli *(APEC), have been found to harbour many fimbriae including Type 1 fimbriae, P fimbriae, curli fibres, S fimbriae, F1C fimbriae, Dr fimbriae, afimbrial adhesins, temperature-sensitive haemagglutinin and many novel adhesin gene clusters that have not yet been characterized. Each of these adhesins is unique due to the recognition of an adhesin-specific receptor, though as a group these adhesins share common genomic organization. A newly identified putative adhesin temporarily termed ExPEC Adhesin I, encoded by gene *yqi*, has been recently found to play a significant role in the pathogenesis of APEC infection, thus making it an interesting candidate for future research. The aim of this review is to describe the role of ExPEC adhesins during extraintestinal infections known till date, and to suggest the idea of investigating their potential role in the colonization of the host gut which is said to be a reservoir for ExPEC.

## Review

Many bacteria have surface appendages of various sizes and appearances called pili and fimbriae. These terms have been used interchangeably. The term "pilus" (plural pili), however, should be reserved for appendages involved in bacterial conjugation, that is the transfer of genetic material, and the term "fimbria" (plural fimbriae) should be reserved for structures concerned with the adhesion of bacteria to various surfaces, including cell surfaces [1]. On average, one to ten conjugative pili and up to more than 400 fimbriae may be present on the surface of a bacterial cell. Conjugative pili are longer than fimbriae and are composed mainly of a pilin protein organized into a tube-like structure, which allows the passage of genetic material during conjugation [1]. Fimbriae have a similar structure but, because they are involved in cellular adhesion, the presence of strain-specific protein sub-units confers a variety of agglutination properties [1].

Pathogenic *E. coli *are the increasing cause of a number of extraintestinal infections, apart from the classical intestinal infections they bring about in humans and animals. Extraintestinal pathogenic *E. coli *(ExPEC), as these pathotypes have now been classified, are responsible for a diverse spectrum of invasive human and animal infections, often leading to septicaemia [2]. Molecular epidemiological analyses have led to the acceptance of ExPEC being distinct from other *E. coli*, as pathogenically versatile, thus reflecting the shared ability of the various ExPEC subsets to overcome or subvert host defences and cause disease at multiple anatomical sites in humans and animals [3]. Unlike non-pathogenic commensal and intestinal pathogenic *E. coli*, ExPEC derive predominantly from *E. coli *phylogenetic group B2, and to a lesser extent from group D [4].

ExPEC, which include Uropathogenic (UPEC), Newborn meningitic (NMEC), Septicaemia associated (SePEC) and Avian pathogenic (APEC) *E. coli*, exhibit considerable genome diversity and possess a broad range of virulence-associated factors including adhesins, toxins, iron acquisition factors, lipopolysaccharides, polysaccharide capsules and invasins [5]. Some of the well known adhesins present among ExPEC strains are Type 1 fimbriae (*fim*), P fimbriae or the pilus associated with pyelonephritis (*pap*), curli fibres (*csg*), S fimbriae or the sialic acid-specific fimbriae (*sfa*), F1C fimbriae (*foc*), Dr fimbriae (*dra*), afimbrial adhesins (*afa*), temperature-sensitive haemagglutinin (*tsh*) and novel adhesin gene clusters many of which remain to be characterized [5-7]. A brief overview of the ExPEC adhesins is given in table 1.

**Table 1:** Adhesins of extraintestinal pathogenic *E. coli*

Adhesin	Gene	Receptor specificity	Cell adherence: *in vitro *and *in vivo *infection models	Reference
Type 1 fimbriae	*fim*	Mannose oligosaccharides (Mono- and Tri-mannose)	Human bladder epithelium, Chicken tracheal and gut explants, colonic and ileal enterocytes	[6,14,15,98,99]
P fimbriae	*pap*	P-blood group antigen-specific glycosphingolipids (α-D-*Galp*-(1-4)-β-D-*Galp*)	Human kidney, colonic and ileal enterocytes	[31,38,99]
Curli	*csg*	Matrix and Plasma Proteins (Fibronectin, Laminin, Plasminogen, H-Kininogen)	Chicken tracheal and gut explants	[51,98,99]
S fimbriae	*sfa*	Neuraminic acid (Sialyl galactosides)	Human bladder and kidney epithelium, Brain endothelium, colonic and ileal enterocytes	[6,99,106-110]
F1C fimbriae	*foc*	Lactosylceramide containing glycolipids	Buccal epithelium, Collecting ducts and distal tubules of human kidney, renal tubulus cells	[6,68-70,73]
Dr fimbriae	*dra*	Dr blood group antigen, Decay accelerating factor (DAF)	Basement membranes of human and canine kidneys, Bowman's capsule, Bladder epithelium, colonic and ileal enterocytes	[75,77,99]
Afimbrial adhesins	*afa*	DAF	Uroepithelial cells	[6,83]
Temperature sensitive haemagglutinin	*tsh*	Haemoglobin, Fibronectin, Collagen IV	Chicken erythrocytes	[90,91]
UPEC trimeric autotransporter adhesin	*upa*	Fibronectin, Laminin	Human bladder epithelial cells	[93]
ExPEC Adhesin I	*yqi*	n.d.	Chicken lung epithelium, Chicken fibroblasts, Canine kidney epithelial cells	[96]

n.d.: Not determined

Interestingly most of the ExPEC adhesins have only been studied with respect to their role in the respiratory tract and urogenital tract of the host pertaining to the extraintestinal infections these pathogens are known to cause. However, several studies hint about the probability of the intestine as a reservoir of ExPEC which suggests that the host could be infected with ExPEC that originate from its own intestinal tract [8-11]. Despite these studies, the role of ExPEC adhesins in the intestinal tract has not yet fully been studied. It is quite possible that these adhesins could in fact play a similar role in the intestinal tract as they do in the respiratory or urogenital tract during ExPEC infection. Therefore we could hypothesize, that if the ExPEC adhesins play a significant role in the adhesion of ExPEC to the intestinal tract, it would increase the chances of ExPEC colonization in the gut of the host, thereby increasing the possibility of an extraintestinal infection under suitable conditions.

### Type 1 Fimbriae

Type 1 fimbrial structures were first noted in early electron microscopic investigations as non-flagellar, filamentous appendages of bacteria. They were first designated "fimbriae" by Duguid in 1955 [12] and termed "pilus" by Brinton almost 10 years later [13]. Since then "pilus" has become a generic term used to describe all types of non-flagellar filamentous appendages and is often used interchangeably with the term "fimbriae". The fimbriae were also classified into types I-V [13] depending on agglutination and binding activity.

Fimbriae were thought to confer the ability to agglutinate erythrocytes and to attach to other cells of varying origin, while several other studies indicated a relation between adhesion and virulence for bacteria inducing infection in relation to a mucous surface [14]. It was also further demonstrated that there was specific binding of one *E. coli *strain to monkey kidney cells, mediated by purified type 1 pili. Moreover, a significant correlation was found between the presence of pili or fimbriae on *E. coli *and the ability of the bacteria to adhere to human urinary tract epithelial cells [14].

Type 1 fimbriae were initially associated with adhesive and pellicle-promoting activities which are inhibited by D-Mannose, that is, they are mannose sensitive [15]. They were described as polymers of pilin subunits that consisted mainly of protein with a high content of hydrophobic amino acids [16]. A single fimbria consists of approximately 100 identical protein subunits [17]. A consensus started developing in the 80's that adhesion is important as a virulence factor in the establishment of infection and that attributes of both the host and the micro-organisms are important in this process [18]. Further research showed that piliated strains of uropathogenic *E. coli *adhered to the polyethylene surface and formed small micro-colonies surrounded by small amounts of glycocalyx, whereas the non-piliated strains adhered only poorly and produced very little extracellular material [18].

The role of type 1 fimbriae and its effect on pathogenicity of APEC infections was first described in 1984, where an observation was made that the presence of adherence pili on the infecting bacteria affected both the number of chicks that developed disease as well as the severity of disease [19].

Over the years these fimbriae have been studied in depth, considering that these organelles provide an ideal model for the study of microbial adherence as stated by Orndorff and Falkow in 1984 [20]. In *E. coli*, 50 - 70% of all strains possess the chromosomally determined type 1 fimbriae [17]. Later studies revealed that four genes designated *fimA*, *B*, *C *and *D *were involved in the synthesis of the fimbriae [21]. The expression of type 1 fimbriae is phase variable that is, the bacteria shift periodically between a fimbriate and non-fimbriate state. It was found that the two regulatory *fim *genes, *fimB *and *fimE *control the phase variation of type 1 fimbriae in *E. coli *[22]. Marc et al. sequenced gene *fimI *of the type 1 fimbrial gene cluster completely and suggested that FimI could possibly constitute a minor fimbrial subunit based on the tryptophan residues in the amino acid composition which are not normally found in the major fimbrial subunit [23]. Three additional genes *fimF*, *fimG *and *fimH *were further characterized and shown not to be necessary for the production of fimbriae but to be involved in the adhesive property and longitudinal regulation of these structures [24]. The receptor-binding adhesin of the type 1 fimbriae was identified, characterized and purified in 1988 and this protein was found to be antigenically conserved among strains with different pilin serotypes, and located at the pilus tip [25,26]. *FimH *was later found to be the gene responsible for the mannose-specific or receptor-specific adhesin of the type 1 fimbriae [27] while *fimC *was found to be the periplasmic chaperone that directs assembly of type 1 pili [28]. Very recently studies involving molecular evolutionary dynamics have shown that there is evidence for strong selection in the type 1 fimbrial adhesin *fimH*, a consequence of which resulted in increased binding of *fimH *to monomannose-containing receptors previously shown to be adaptive for Uropathogenic *E. coli*, and which also correlates with increased adhesion to vaginal epithelial cells [29].

The type 1 fimbriae have thus been structurally and functionally characterized extensively over decades. There is ample evidence that type 1 pili play a significant role as mediators of attachment by *E. coli *infections, particularly UPEC infections [14]. *FimH *being the receptor specific adhesin, has been utilized as a vaccine candidate in previous studies involving urinary tract infection caused by UPEC. One such study showed that immunization with *fimH *reduced *in vivo *colonization of the bladder mucosa by more than 99 percent in a murine cystitis model, and immunoglobulin G to *fimH *was detected in urinary samples from protected mice. Furthermore, passive systemic administration of immune sera to *fimH *also resulted in reduced bladder colonization by UPEC [30]. The type 1 fimbriae best studied in *E. coli*, therefore, are of tremendous importance in the pathogenesis of Gram negative bacteria.

### P fimbriae

The P fimbriae are morphologically indistinguishable from the type 1 fimbriae, however, they recognize and bind to the α-D-Gal*p*-(1-4)-β-D-Gal*p *carbohydrate sequence occurring in the series of P-blood group antigen-specific glycosphingolipids [31] and hence the name. The genes encoding the P pilus type were termed the *pap *genes or pyelonephritis-associated pili genes since these were typical of strains isolated from human urinary tract infections [32]. *PapA *was described to be the structural gene for the P fimbriae monomer [33], that is, these pili, which are hair-like appendages consist of helically arranged subunits of the protein *papA*. The protein *papG *is the digalactoside-specific adhesin and through immuno-electron microscopy it was found that the P pili are heteropolymers composed of the major pilin, *papA*, the minor pilins, *papE *and *papF*, and the adhesin *papG *[34], the last three proteins being located at the tip of the pilus. These findings were further confirmed by Lund et al [35], who showed that the major subunit *papA *is not required for binding, but *papF *and *papG *are essential for adhesion.

There are several different serotypes of P fimbriae, which are said to differ in their serological differences due to structural variation within the central domain of the major pilus subunit [36]. P pilus biogenesis has been studied extensively unravelling the function of each gene of the *pap *gene cluster or operon [37]. A model of the pilus biogenesis which involves 11 genes organized in the *pap *gene cluster on the chromosome in clinical isolates expressing P pili has also been previously described [38]. The PapD chaperone interacts with each pilus protein subunit forming assembly competent complexes which are targeted to the PapC outer membrane assembly protein required for P pilus biogenesis. PapA subunits pack into a right-handed helical rod. PapH incorporation terminates polymerization of PapA and anchors the pilus to the cell. PapE subunits form open helical fibres called tip fibrillae.

The PapG adhesin, positioned at the distal end of the fibrillum, mediates binding to the Galα (1-4) Gal receptor determinant [38] or the globo series of glycolipids in the human kidney. PapG recognition of the galabiose receptor is thought to be a prerequisite for pyelonephritis [39].

It has been reported that more than 80% of all pyelonephritogenic strains of *E. coli *express P fimbriae, which are recognized as a key determinant in promoting the virulence of *E. coli *in urinary tract infection (UTI) [40]. Furthermore, P-fimbriated *E. coli *interact poorly with neutrophils and resist their bactericidal actions *in vitro*. P fimbriae are thought to play a role by means of their PapG adhesin, which occurs in three molecular variants: PapGI, PapGII and PapGIII [41]. Studies involving different animal models of ascending UTI have found a variable role for P fimbriae, in particular PapGII-mediated adherence, a variant of the PapG adhesin, in the colonization of the mammalian kidney. Molecular epidemiological studies have shown that allele III of PapG is usually the predominant variant among *E. coli *isolates from women and children with cystitis, while the PapGII variant is associated with pyelonephritis and bacteremia in humans [8,10]. Overall it appears that there is a subtle role for P fimbriae in mediating adherence to uroepithelial cells *in vivo *and establishing a robust inflammatory response during renal colonization, which in turn contributes to kidney damage during acute pyelonephritis [8,42]. It is now fully established, that these P pili adhesive organelles are critical virulence factors, that mediate the recognition of and attachment to tissues of the kidney by the pathogen during UTI [43]. It has also been previously shown that P fimbriae utilize the toll-like receptor 4 (TLR4)-dependent pathway to trigger mucosal inflammation [44].

The P fimbriae are not only restricted to Uropathogenic *E. coli *(UPEC) causing UTI, but are also prevalent in Newborn meningitic *E. coli *(NMEC) and Avian pathogenic *E. coli *(APEC) strains [5,45,46]. The importance of P fimbriae among APEC has gained a lot of interest over a period of time. It has been previously observed that the *pap *positive genotype was associated more frequently with pathogenic isolates from septicaemic chickens than from healthy chickens, suggesting its function during septicaemic infection [47]. The potential of P pili as a vaccine candidate has also been studied and it has been observed that vaccination with Gal-Gal pili or the P fimbrial vaccines prevented pyelonephritis by piliated *E. coli *in a murine model as well as in monkeys [48,49].

### Curli Fibres

The name curli was proposed in 1989 to a third class of *E. coli *surface organelles in addition to the flagella and fimbriae, which were found to be coiled surface structures composed of a single type of subunit, the curlin, which differs from all known pilin proteins and is synthesized in the absence of a cleavable signal peptide [50]. Most natural isolates of *E. coli *carry a transcribable curli gene, *crl*, however only certain strains are able to assemble the subunit protein into curli [50].

Curli bind several matrix and plasma proteins such as fibronectin, laminin, plasminogen, tissue plasminogen activator, and H-kininogen [51]. Curli fibres are encoded on the *csg *(curlin subunit gene) gene cluster, comprised of two differently transcribed operons, one which encodes the *csgB*, *csgA *and *csgC *genes, and a second which encodes *csgD*, *csgE *and *csgG *[52]. Curli in *E. coli *consist of polymers of a single 15-kDa protein encoded by the subunit gene *csgA *and production of the curli fibres requires expression of both operons [51]. Assembly of curli fibres involves extracellular self-assembly of the subunit *csgA*, dependent on a specific nucleator protein *csgB*. *CsgD *is a transcriptional activator essential for expression of the two curli fibre operons, while *csgG *is an outer membrane lipoprotein involved in extracellular stabilization of *csgA *and *csgB *[52]. The expression of genes coding for curli is complex and involves several control elements, such as H-NS, RpoS and OmpR which results in a great reduction in the expression of curli fibres at temperatures higher than 30°C and at high osmolarity in most strains [52]. Recently a novel regulator, termed MlrA was found to be required for curli production and extracellular matrix formation [53]. The ability of curli polymers to specifically interact with numerous human proteins such as the matrix proteins fibronectin and laminin, and proteins of the fibrinolytic and contact-phase systems, facilitates the adaptation of curli-expressing bacteria to different niches in the infected host [51]. It has further been shown that curliated *E. coli *in human plasma absorbs plasminogen and tissue plasminogen activator, leading to the formation of proteolytically active plasmin which may promote bacterial spreading through tissue degradation [51].

Polymerization of curlin to fimbriae-like structures (curli) on the surface of *E. coli *differs from the prevailing model of fimbrial assembly, in that, it occurs extracellularly through a self-assembly process depending on a specific nucleator protein [54]. Curli polymers are formed as a result of a conformational change of soluble *csgA *initiated by an interaction with a nucleating *csgB *protein and such an induced conformational change might involve a conversion from a partially disordered structure in the monomeric state to readily ordered secondary structures in the polymeric state [54].

Studies on the pathogenic role of curli in avian pathogenic *E. coli *infections have also been carried out, and there is evidence that haemagglutination activity, fibronectin binding and curli production are co-expressed in an APEC strain and haemagglutination and fibronectin binding are recognized as virulence factors that may be important in the adherence of pathogens to host surfaces [55]. In another study, it was seen that 99% *E. coli *isolated from diseased birds possessed the *csgA *gene responsible for curli biosynthesis [56]. Furthermore curli fibres were found to be essential for the internalization of bacteria causing avian septicaemia as seen *in vitro *[52].

### S Fimbriae

The S fimbriae were discovered as a group of fimbriae among pyelonephritogenic *E. coli *strains which recognized neuraminic acid (sialic acid) - containing structures other than mannosides or P antigens on human erythrocytes [57] and were termed the S fimbriae based on their receptor specificity, that is, their specific binding to sialyl galactosides [58]. Morphologically, S fimbriae are similar to type 1 or P fimbriae of *E. coli*, that is, they are 1 to 2 μm in length, around 5 to 7 nm in diameter and their subunit size is equal to that of type 1 fimbriae of *E. coli *[58]. The S-fimbrial adhesins (Sfa) were reported to be most often found among meningitis- and sepsis-associated *E. coli *isolates [59].

The *sfa *genetic determinant (6.5 kb) for these fimbriae was cloned and found to code for at least seven *sfa*-specific gene products [59]. This determinant represents a cluster of genes with a homogeneous genetic structure and consists of different regions involved in the production of the fimbriae and the adhesin, the biogenesis of fimbriae, and the control of transcription [60]. *SfaS*, the minor subunit of the S fimbriae, a 14 kDa protein, localized at the distal end of the *sfa *gene cluster, was identified as the sialic acid - binding adhesin [61]. The entire *sfa *gene cluster consists of *sfaA*, a major subunit protein of 16 kDa and three minor subunit proteins *sfaS *of 15 kDa, *sfaG *of 17 kDa and *sfaH *of 29 kDa, which together form the *sfa *complex [62]. Expression of the *sfa *determinant is dependent on several environmental conditions, such as temperature, osmolarity, and the presence of glucose, while at the molecular level, regulation of the *sfa *determinant is mediated by two regulatory proteins *sfaB *and *sfaC *[63].

In a study on the prevalence of the S fimbriae among ExPEC strains, it was observed that 50% UPEC, 24% NMEC and 9.2% APEC strains harboured the *sfa *genes [5]. In another study 79% of the septicaemic and diarrheic *E. coli *isolates from pigs tested were found to be positive for S fimbriae, while yet another study showed that the prevalence of the S fimbriae in human and avian ExPEC isolates of a certain phylogenetic group was 100% and 97% respectively [46,64]. Similarly various genotyping studies have also reported the prevalence of S fimbriae among ExPEC isolates [9,10,65].

It has been shown that S-fimbriated bacteria and the purified S fimbriae bind specifically to human epithelia, for example, the vascular endothelium in both large vessels of kidney tissue, the capillary endothelium in the interstitium and the visceral epithelium of the glomerulus which are known to have a sialic acid coating [66]. An important observation is that S fimbriae occur in some pyelonephritogenic *E. coli *strains but are mainly associated with strains causing neonatal sepsis and meningitis [66]. S fimbriae have also been shown to bind the extracellular matrix components of fibronectin and laminin and sialoglycoproteins on brain microvascular endothelial cells, an interaction that may explain migration across physiological barriers [6].

A recent study revealed the identification of a new fimbrial cluster of the S-fimbrial adhesin family, which was termed AC/I (avian *E. coli *I) or *fac *(fimbriae of avian E. coli strains) [67]. These fimbriae did not haemagglutinate red blood cells but were shown to adhere to avian tracheal cells. Furthermore long-range mapping with specific DNA probes showed that these fimbriae were related to S fimbriae [67].

### F1C Fimbriae

A single F1C fimbria is a thin, 7-nm-wide, approximately 1 μm long surface polymer whose structure closely resembles that of type 1 fimbriae [68]. F1C fimbriae, with subunits of about 17K, confer no haemagglutination to erythrocytes from humans, oxen, horses, guinea-pigs or chickens; however, they adhere to buccal epithelial cells [69]. Although these fimbriae are not haemagglutinating, they contribute to the adhesive properties of UPEC strains, in that they mediate specific adherence to the collecting ducts and the distal tubules of the human kidney [70], as well as to cultured renal tubulus cells [68]. The *foc *(fimbriae of serotype 1C) gene cluster is involved in the synthesis of F1C fimbriae. This gene cluster which has been cloned and studied shows that six genes are involved in the biogenesis of F1C fimbriae, including *focA *which encodes the major fimbrial subunit, *focC *which encodes a product that is indispensable for fimbria formation, *focG *and *focH *which encode minor fimbrial subunits, and *focI *that encodes a protein which shows similarities to the subunit protein *focA *[70].

The F1C fimbriae (*foc*) genetic determinant is related to the S fimbriae (*sfa*) genetic determinant, both of which show a high degree of homology, in that, they show similarities in their DNA sequence composition and exhibit common epitopes on their corresponding fimbrial proteins; however, the Sfa and F1C antigens differ in their receptor specificities [71]. It has been shown that *foc*-specific gene products are able to produce a wild-type phenotype in *sfa *insertion mutants and that hybrid DNAs consisting of *sfa*- and *foc*-specific sequences code for intact fimbriae after transformation into non fimbriated *E. coli *strains [71].

Until recently the exact receptor specificity of the F1C fimbriae was not known; however, in 2000, glycolipid receptors for purified F1C fimbriae were identified. TLC (thin-layer chromatography) fimbrial overlay analysis revealed the binding ability of purified F1C fimbriae only to glucosylceramide (GlcCer), β1-linked galactosylceramide 2 (GalCer2) with non hydroxyl fatty acids, lactosylceramide, globotriaosylceramide, paragloboside (nLc_4_Cer), lactotriaosylceramide, gangliotriaosylceramide (asialo-GM_2 _[GgO_3_Cer]) and gangliotetraosylceramide (asialo-GM_1 _[GgO_4_Cer]) [72]. It has also been suggested that the disaccharide sequence GalNAcβ1-4Galβ of asialo-GM_2 _(GgO_3_Cer) which is positioned internally in asialo-GM_1 _(GgO_4_Cer) is the high-affinity binding epitope for the F1C fimbriae [72]. It was further reported that F1C fimbriated bacteria selectively interact with two minor glycosphingolipids isolated from rat, canine, and human urinary tract, and comparison of the binding-active compounds with reference glycosphingolipids revealed that the receptor specificity is dependent on the ceramide composition [73].

### Dr Fimbriae

Vaisanen-Rhen et al. [74] originally described a mannose-resistant P blood group-independent haemagglutinin which was expressed by a number of UPEC strains belonging to serogroup O75; accordingly, this adhesin was named O75X [75]. Nowicki et al. showed that the Dr blood group antigen, a component of the IFC (Inab-Freiberger Cromer) blood group complex, is the receptor for the O75X fimbrial-like adhesin and the molecule recognized by the Dr haemagglutinin is a chloramphenicol-like structure [76]. The name Dr haemagglutinin for the O75X fimbrial-like adhesin was therefore proposed [76]. It was observed that the Dr blood group substance was found in the tubular basement membrane and Bowman's capsule of the human kidney [76] and Dr adhesins have been shown to bind preferentially to basement membranes of human and canine kidneys, Bowmans capsule and to a lesser extent to the bladder epithelium [75].

The Dr fimbriae or O75X fimbriae are chemically very similar but morphologically different from typical *E. coli *fimbriae, and electron microscopy has revealed that the purified proteins were shown to be arranged in a coil-like structure which consists of subunits with an apparent molecular mass of about 15 kDa [77]. The Dr adhesin-encoding operon was identified and termed *dra *of which four genes, *draA*, *draC*, *draD *and *draE *are required for full expression of the mannose resistant haemagglutinin phenotype [75]. *DraE *of the Dr operon encodes the major structural subunit that compose the respective fimbrial appendages and is also the adhesive subunit for the DAF (decay accelerating factor) receptor [78]. The products of the *draB *and *draC *genes exhibit similarity to chaperone-usher proteins belonging to the superfamily of PapD like chaperones [79].

A number of studies have assessed the role of Dr fimbriae in the pathogenesis of extraintestinal pathogenic *E. coli*. It has been reported that *E. coli *with Dr fimbriae persisted in the kidney tissue and were associated with significant tubulointerstitial nephritis, whereas an *E. coli *mutant without Dr fimbriae was gradually cleared from kidney tissue which displayed significantly less pathology [80]. It was also observed that infections during pregnancy with *E. coli *bearing adhesins of the Dr family may pose a threat for patients due to bacterial invasive potential and pregnancy-associated up-regulation of DAF receptor [81].

Immunization of mice with the *E. coli *Dr fimbrial antigen reduced mortality associated with an experimental urinary tract infection due to a homologous strain bearing the Dr adhesin, while immune sera with high titers of anti-Dr antibody inhibited bacterial binding to the bladder and kidneys but did not affect the rate of renal colonization [82]. Dr fimbriae have been found to be prevalent among APEC (1.3%), UPEC (6.1%) and NMEC (3.8%) isolates; however, in a lower percentage as compared to type 1 fimbriae, P fimbriae or S fimbriae [5].

### Afimbrial adhesins

More than 20 years ago, it was observed that 10% of the *E. coli *strains, which agglutinated human erythrocytes in the presence of D-mannose, also termed mannose-resistant haemagglutination (MRHA), did not show any fimbriae and still adhered to uroepithelial cells, suggesting the existence of afimbrial adhesins [83]. It was also found that about 6.7 kb of DNA were required for the expression of the MRHA of human erythrocytes and to confer adhesion, and that this binding function was mediated by a 16 kDa protein named AFA-I [83].

The 6.7 kb insert expresses five polypeptides of molecular mass 13 kDa, 16 kDa, 18.5 kDa, 30 kDa, and 100 kDa, encoded, respectively, by the *afaA*, *afaE*, *afaD*, *afaB *and *afaC *genes which are localized and belong to the same transcriptional unit [84]. The *afaE *gene encodes the adhesin or haemagglutinin AFA-I polypeptide, the *afaB *gene is also required for MRHA expression, however, does not play an obvious role in the biosynthesis or the maturation of the AFA-I haemagglutinin, while the *afaC *gene codes for a polypeptide synthesized as a precursor and its gene product is transported through the cytoplasmic membrane by means of a signal sequence [84]. Purification and characterization of the afimbrial adhesin AFA-I showed that it exists on the bacterial surface and free as a macromolecular aggregate in the supernatant of spent culture medium, and is composed of a single, repeating 16 kDa polypeptide subunit [85]. Transformation of non adherent recipient pyelonephritic strains with recombinant plasmids carrying the *afa-I *operon confers binding specificities and biochemical properties different from those observed with strains expressing type 1, P fimbriae and S fimbriae [86].

It was later demonstrated that there exist gene clusters structurally related to the first *afa *operon which was described, but which encoded antigenically distinct afimbrial adhesins; experiments demonstrated that all the *afa *gene clusters harboured a highly conserved 4.1 kb DNA segment carrying the *afaB*, *afaC *and *afaD *genes and revealed heterogeneity for the *afaE *sequences [87]. It was therefore proposed that there exist at least four different *afa *operons, *afa-1*, *afa-2*, *afa-3 *and *afa-4 *which encode variable adhesins designated AFA-I, AFA-II, AFA-III and AFA-IV respectively [87]. AFA-I and AFA-III belong to a family of haemagglutinins which also include the Dr fimbrial adhesin, and this heterogeneous adhesin family is referred to as the Dr family [87]. Two *afa *operons, designated *afa-7 *and *afa-8 *found in bovine isolates were cloned and analyzed, and *afa-8 *was found to be widespread among bovine pathogenic *E. coli *strains associated with diarrhoea and septicaemia [88]. Prevalence studies have shown that afimbrial adhesins occur among APEC (1.3-8.2%), UPEC (6.1-12.6%) and NMEC (3.8-25.6%) isolates [5,65].

### Temperature sensitive haemagglutinin

A mannose-resistant haemagglutinin of an avian pathogenic *E. coli *(APEC) isolate was identified, which was found to be best expressed at lower temperatures [89]. Haemagglutination activity was highest when cells were grown at 26°C and lower in cells grown at 37°C, while cells grown at 42°C lacked activity. This temperature-dependent haemagglutination phenotype was termed Tsh for temperature sensitive haemagglutinin [89]. The gene responsible for the Tsh phenotype, *tsh*, was cloned and characterized and found to confer a haemagglutination-positive phenotype to *E. coli *K-12 strains [89]. It was found that *E. coli *K-12 strains containing a recombinant *tsh *gene produce two proteins, a 106 kDa extracellular protein and a 33 kDa outer membrane protein, and were able to agglutinate chicken erythrocytes [90]. Further studies revealed that Tsh is synthesized as a 140 kDa precursor protein, whose processing results in a 106 kDa passenger or secreted domain and a 33 kDa β- barrel domain [91,92]. The role of Tsh during pathogenesis of APEC infections has been studied. It was demonstrated that out of 300 avian *E. coli *isolates examined for the prevalence of the *tsh *gene, half of the isolates were *tsh *positive and *tsh *was specifically more frequent in high-lethality isolates compared to low-lethality isolates [92]. In another study it was shown that the *tsh *gene was prevalent in more than 50% of APEC, 4.5% UPEC and 11.5% NMEC isolates tested [5]. It was further seen that in the *tsh *positive strains examined, *tsh *was always plasmid encoded and was linked to colicin V genes when they were present on the same plasmid [92]. In an additional study it was also reported that purified Tsh secreted domain is capable of adhering to red blood cells, haemoglobin, and the extracellular matrix proteins fibronectin and collagen IV [91].

### Novel adhesin gene clusters

With the availability of whole genome sequences for crucial pathogens, including many ExPEC prototypic isolates, many novel genes, including those encoding putative adhesins, have been found to be present on the genome of these strains and it has become clear that a single strain produces many different adhesins at one time or the other. A novel trimeric autotransporter adhesin UpaG was recently identified through a reverse vaccinology approach as a potentially protective antigen against ExPEC and characterized for its role in ExPEC adhesion [93,94]. UpaG is an adhesin located on the cell surface, exported there by virtue of a C-terminal β-domain, and mediates the aggregation of *E. coli *as well as its adhesion to abiotic surfaces, T24 bladder epithelial cells, and extracellular membrane proteins [93].

Additionally, the sequencing of a prototypic cystitis strain UTI89 revealed that the strain contains at least ten different chaperone-usher adhesin systems including *fim *and *pap *which still remain the only two adhesins best characterized [6,95]. Other putative adhesins like *auf*, *yad*, *yqi*, *yeh *and *fml *have been identified in UTI89; however, all except for *yqi*, have yet to be characterized for their role in pathogenesis. The adhesin, Yqi, has been now found to play an important role in colonization, the first step of pathogenesis, during APEC infection [96].

### ExPEC adhesin I (yqi) - a novel fimbrial adhesin of ExPEC

Recently a new fimbrial adhesin encoded by gene *yqi *was identified in an APEC strain IMT5155 (O2:K1:H5) via signature-tagged mutagenesis in a chicken lung colonization model of infection [96,97]. Its gene product has been temporarily designated ExPEC Adhesin I (EA/I) until the specific host receptor for this adhesin can be identified, which would permit better classification and nomenclature of the novel adhesin in the future. ExPEC Adhesin I has now partially been characterized and it has been shown that deletion of the adhesin gene *yqi*, resulted in reduced colonization ability by APEC strain IMT5155 both *in vitro *and *in vivo*. The adhesin gene *yqi *is located on a 4,975 bp gene cluster. Genomic organization of this gene cluster is similar to the genomic organization of the P fimbrial *pap *operon, in that, the putative outer membrane usher precedes the periplasmic chaperone which is followed by the adhesin gene. A conserved hypothetical protein gene precedes the usher in the *yqi *gene cluster, which may eventually code for the adhesin subunit protein; however, this still needs to be proven. Cloning of the 4,975 bp adhesin gene cluster in an afimbriate *E. coli *strain *in vitro*, led to the expression of short fimbrial like appendages protruding out of the bacterial outer membrane as observed by electron microscopy.

The adhesin encoding gene *yqi *was found to be prevalent among ExPEC isolates including APEC, UPEC and NMEC by more than 50 percent, and absent in all of the intestinal pathogenic *E. coli *strains tested, thereby validating the designation of the adhesin as ExPEC adhesin I. In addition, prevalence of the adhesin was most frequently associated with the B2 phylogenetic group and ST95 complex of the multi locus sequence typing (MLST) scheme http://mlst.ucc.ie/mlst/mlst/dbs/Ecoli/, with evidence of a positive selection within this highly pathogenic complex [96].

### Role of ExPEC adhesins in the gut

So far, the functional role of adhesins in the colonization of the intestinal tract by ExPEC is inadequately understood. Limited studies have dealt with the aspects of intestinal colonization by ExPEC, being extraintestinal pathogens, thereby focussing on infection models involving extraintestinal anatomical sites or cell lines in order to study the adhesion and colonization abilities of these pathogens better. Nevertheless, in one study, the role of type 1 fimbriae and curli in an APEC strain was even studied with respect to colonization of the gut of the chicken suggesting the role of these adhesins in the intestinal tract [98]. In a separate study type 1, P, S and Dr fimbriae, were found to mediate binding to colonic and ileal enterocytes [99]. Recently Schierack et al. reported the isolation of a haemolytic *E. coli *clone that dominated the coliform flora of piglets, and which did not harbour any virulence determinants typical for intestinal pathogenic *E. coli *isolates from swine, but had a virulence gene profile very similar to ExPEC and harboured type 1 and P fimbriae, besides curli and temperature sensitive haemagglutinin [100]. The authors further suggested the potential role of ExPEC virulence associated genes, including adhesins, in the successful intestinal colonization ability in piglets.

Apart from ExPEC, intestinal *E. coli *also harbour adhesins like type 1 fimbriae, curli and F1C fimbriae, and the function of these adhesins in intestinal colonization has been fairly well studied. Recently the F1C fimbriae were found to play an important role in intestinal colonization by a commensal strain *E. coli *Nissle 1917 [101], while curli were found to be responsible for increased adherence of Shiga-toxin producing *E. coli *strain to a colonic cell line [102].

Taken together these studies signify the importance of ExPEC adhesins in the gut of the host, providing a sound basis for investigating their role in intestinal colonization further.

## Conclusion

The group of *E. coli *known as the extraintestinal pathogenic *E. coli *(ExPEC) show great variety in the fimbrial adhesin systems they possess. The presence of multiple pilus systems likely confers niche-adaptive advantages, and the combination of receptor specificity and tissue-specific receptor production will ultimately determine the site of action for a given pilus during infection. ExPEC harbour many known and unknown adhesin systems. Ahmed et al. previously reported that bacterial genome fluidity could be exploited for diagnostic and health-care applications, for example, the presence or absence of virulence genes, or the expression of functional virulence factors could be used as diagnostic markers and antigens of markers of infection [103]. Therefore, understanding and functionally characterizing the roles of putative adhesins in pathogenesis will most likely provide new targets for therapeutic intervention in the diagnosis, treatment and prevention of ExPEC infection.

Most adhesins function by binding to a specific receptor on the host tissue during infection as already described. Therefore, a single pathogen may make use of multiple adhesins, with affinities to different specific receptors which enable the pathogen to adapt to different host tissues during initiation of infection. The receptor for the newly identified ExPEC adhesin I is still unknown, and the identification of such a receptor would indeed be of great value in future prevention therapies, for example, by blocking of the receptors to prevent the pathogen from attaching to host surfaces. In addition it will also allow for appropriate nomenclature of this adhesin in the future.

Since for most bacteria, the first encounter with their host involves attachment to a eukaryotic cell surface, which results in colonization of the host prior to disease, induced antibody responses at the mucosal surface could prevent attachment and abrogate colonization [104]. The ideal target, therefore, for such antibodies are the surface proteins or adhesins which mediate microbial attachment to host tissue [105], making them extremely good vaccine candidates. An important aspect here is that the intestine is said to be a reservoir for ExPEC, suggesting that ExPEC would initially colonize the intestinal tract which could eventually then lead to an extraintestinal infection given the right conditions. In this case, it might be interesting to find out whether the ExPEC adhesins play a role in colonization of the intestinal tract by these pathogens. To date, there are to the best of our knowledge only a handful of studies describing the role of ExPEC in the gut as already described; however, this could be an interesting study area, and adhesin vaccines could be developed in future that may be more effective in the intestinal tract, an interesting facet that could be used for the prevention of extraintestinal infection. Thus, vaccination studies with newly identified adhesins like ExPEC adhesin I could indeed prove vital and promising in the future in order to subsequently prevent ExPEC infections that are still a threat to many.

## Competing interests

The authors declare that they have no competing interests.

## Authors' contributions

EA drafted and wrote the manuscript. CE and LHW were involved in revising the manuscript critically for important intellectual content. All authors read and approved the final manuscript.
